# A Case of Recurrent Erysipelas Caused by *Streptococcus mitis* Group

**DOI:** 10.1155/2018/5156085

**Published:** 2018-05-30

**Authors:** David Nygren, Bo Nilson, Magnus Rasmussen

**Affiliations:** ^1^Division of Infection Medicine, Department for Clinical Sciences Lund, Medical Faculty, Lund University, Lund, Sweden; ^2^Clinical Microbiology, Region Skåne Labmedicin, Lund, Sweden; ^3^Division of Medical Microbiology, Department of Laboratory Medicine, Medical Faculty, Lund University, Lund, Sweden

## Abstract

The aetiology of erysipelas remains poorly defined though beta-haemolytic streptococci are considered as the main causative pathogens. We describe a case of a 70-year-old woman with recurrent erysipelas in her left arm due to infection with streptococci of the mitis group. Her past medical history includes lymphoedema of the left arm secondary to lymph node dissection due to breast cancer surgery. On seven different occasions during a decade, she has presented a clinical picture of erysipelas and in three of them with *Streptococcus mitis* group bacteraemia. The results indicate that two cases were caused by *Streptococcus mitis* and one case was caused by *Streptococcus oralis*. This is, to our knowledge, the first reported cases of *S. mitis* and of *S. oralis* as the causative agents of erysipelas.

## 1. Introduction

The aetiology of erysipelas (superficial cellulitis) remains poorly defined as causative bacteria are isolated only in a minority of patients. However, beta-haemolytic streptococci (BHS) are believed to be responsible in most cases [1]. Bacteraemia is rare in erysipelas, and a systematic review of five studies on erysipelas demonstrated that only 4.6% of patients had positive blood cultures, of which a majority grew BHS [2]. We have previously reported bacteraemia in 9% of blood cultures from patients with erysipelas, of which a large majority (86%) grew BHS [3]. Cultures from needle aspirates or punch biopsies of the inflamed skin are negative in a majority of cases [4, 5], and even using sensitive PCR-based methods, causative pathogens are seldom identified [6, 7]. In support for BHS aetiology of erysipelas, one study using immunofluorescence identified BHS in 19 of 27 erysipelas cases [8]. Serological studies also support BHS aetiology in a share of the cases, but results have been somewhat conflicting [4, 8–10]. Risk factors to contract erysipelas as well as for it to recur are known to involve lymphoedema, a site of entry, overweight, and venous insufficiency [11, 12]. Since the aetiology of erysipelas is not always clear, this report, describing erysipelas caused by *Streptococcus mitis* group, is of particular interest. *S. mitis* and *S. oralis* are oral commensals which are described to be increasingly resistant towards penicillin [13–17]. Bacteria of the *S. mitis* group are previously unidentified pathogens of erysipelas yet a known cause of infective endocarditis [18]. The mitis group of viridans streptococci comprises several species, which can be determined to the species level or to the subgroup level using matrix-assisted laser desorption ionization/time-of-flight mass spectrometry (MALDI-TOF MS). Differentiating *S. mitis*, *S. oralis*, *S. pneumoniae*, and *S. pseudopneumoniae* of the *S. mitis* group remains difficult using MALDI-TOF MS [19]. Recently, the performance of separating out and identifying *S. pneumoniae* has been improved by combining a better MALDI Biotyper database with a new algorithm for weighted list (score) [20]. By assigning specific peak combinations in the mass spectra that are associated with specific species in the *S. mitis* group, one may further improve species determination [21, 22].

## 2. Case Report

A 70-year-old woman presented in November 2017 to the Emergency Department at Skåne University Hospital, Sweden, due to the rapid onset of fever, shivers, and a suspected skin infection. She had a previous medical history of left-sided ductal breast cancer with lymph node involvement in 1999, which was treated chronologically with neoadjuvant chemotherapy, partial mastectomy, axillary lymph node dissection, and radiation therapy. In addition, in 2001, a right-sided localised ductal breast cancer *in situ* was identified and was treated surgically with a partial mastectomy. Secondary to her lymph node dissection, she developed lymphoedema of her left arm, which had been continuously treated with compression stockings. The patient was on treatment with an ACE inhibitor and a beta-blocker due to hypertension, and in addition, she had a known systolic murmur, characterized as physiological, as transthoracic echocardiographs in 2011 and 2017 were normal. Since her surgery in 1999, on a total of six occasions prior to her last and seventh visit, of which the first episode occurred in 2008, she had been treated for erysipelas in her left upper arm. The presentation had always been sudden with spiking fever and erythema spreading in approximately the same localisation. Interestingly, on all three out of the three occasions where a blood culture has been drawn on presentation with erysipelas, the cultures have shown growth of a bacterium belonging to the *S. mitis* group. These first two isolates also had similar MIC values for penicillin of 0.064 and 0.125 mg/L, for vancomycin of 0.25 and 0.5 mg/L, and for gentamicin of 2 and 2 mg/L (Table S1). In addition, they were both sensitive to clindamycin.

On the present visit, she once again had a sharply demarcated, warm, swollen, and painful erythema measuring approximately 7 × 15 cm in the lymphoedematous area on her left upper arm. No local portal of bacterial entry was found. Vital parameters showed a temperature of 38.0°C, respiratory rate of 16 breaths/min, O_2_ saturation of 96% on room air, heart rate of 80 beats/min, and blood pressure of 120/70 mmHg. On physical examination, a grade II systolic murmur was heard with punctum maximum I2 dexter. She had no signs of septic emboli, oral examination showed no signs of infection, and examination of lymph nodes was normal. Possibly due to her quick presentation, that is, less than 6 hours from the onset of symptoms, her laboratory results were normal with a white blood cell count of 8.4 ∗ 10^9^/L, platelets of 263 ∗ 10^9^/L, and hemoglobin of 147 g/L. Her CRP was 12 mg/L. She was clinically diagnosed with erysipelas, and due to previous bacteraemia with the *S. mitis* group in relation to erysipelas and the presence of a systolic murmur, blood cultures were drawn and she was treated with one dose of intravenous penicillin (3*g*≈5 million IU) followed by an oral penicillin (1*g*≈1.6 million IU) three times daily, for seven days. Once again, now for the third time, the two blood cultures showed growth of a bacterium belonging to the *S. mitis* group. The MIC value for penicillin was 0.125 mg/L, for vancomycin 1 mg/L, and for gentamicin 16 mg/L (Table S1). Similar to the two previous isolates, it was also sensitive to clindamycin. Her treatment was prolonged for 10 days, and a follow-up visit was arranged. Repeat blood cultures were drawn 14 days after discontinuation of antibiotics and they were negative. To prevent further infections, she has once again been referred to the lymphoedema outpatient clinic as well as to the dentist office. On follow-up, thereafter, the patient had no sequelae to her infection, and she gave informed consent for this case report to be published.

The three blood isolates, one analysed in 2015 and two in 2017 (15 and 8 months apart), were initially subgrouped to *S. mitis/S. oralis/S. pseudopneumoniae* of the *S. mitis* group by combining the MALDI-TOF MS results (MALDI Biotyper, Bruker) with the information that the three stains were resistant to optochin. To allow a more detailed comparison, the three stored isolates were reanalysed and now ethanol/formic acid extractions were performed on the strains, and the updated and improved Bruker MALDI Biotyper database (DB-7311 MSP Library) was used for the MALDI Biotyper analysis. In addition to the standard log (score), weighted list (scores) was also calculated [20]. *S. mitis* was the best match for both the first and second isolates when both log (score) and list (score) were calculated. For the third isolate, the best match was *S. oralis* for both types of scores (Table S1). Next, the mass spectra of the three isolates were inspected manually. All three strains showed the specific peak 6839.1 m/z which is associated with *S mitis* and *S. oralis* strains, but only the third isolate showed the specific peak 5822.5 m/z which is associated with *S. oralis* (Table S1) [21]. In addition, no peak profiles typical for *S. pneumoniae* and *S. pseudopneumoniae* could be detected in the three isolates [21, 22]. These results further support that the first two isolates are *S. mitis* and the third isolate is *S. oralis*. Many differences were seen in the mass spectra of the third isolate (*S. oralis*) compared to the first two (*S. mitis*). On the other hand, no clear differences in the spectra between the first and second isolate could be seen, and one can therefore not exclude that they belong to the same clone.

## 3. Discussion

Erysipelas is a common skin infection described to be caused by BHS [1]; nevertheless, the causative agent is seldom verified [2, 4–6]. This case report demonstrates a recurrent erysipelas infection due to viridans streptococci, and our results indicate that two specific species of the *S. mitis* group, *S. mitis* and *S. oralis,* caused the infection at different occasions. To our knowledge, *S. mitis* and *S. oralis* causing erysipelas has never been previously described. Since our patient had lymphoedema of her arm secondary to lymph node dissection, previously found to be the most significant risk factor of recurring erysipelas in the upper extremities [11], we hypothesize that the recurrence of infection could be due to a chronic colonization of the skin and mouth by the *S. mitis* group bacteria. The results suggest that the two first recurring infections were both caused by *S. mitis*, and they showed very similar mass spectrum and antibiotic susceptibility profiles; therefore, it seems likely that the first two episodes were caused by the same clone. Furthermore, a possible explanation could be that minor skin abrasions related to taking on and off compression stockings could act as a bacterial portal of entry through the skin.

In addition, due to the recurring infections, it could be argued that there could possibly be a problem of source control, since the *S. mitis* group is also a known cause of endocarditis [18]. However, the recurrences have been far apart and the treatment has always been uncomplicated without treatment failure or signs of endocarditis on echocardiography.

The three isolates of the *S. mitis* group were susceptible to the empirical treatment with penicillin, which is the recommended treatment for erysipelas due to its effect on BHS [1]. Should *S. mitis* and *S oralis* prove to be emerging pathogens of erysipelas, this might pose problems since studies on antimicrobial susceptibility testing generally demonstrate penicillin resistance among the mitis group [13–15, 17], and in one study as high as 60% [16].

In summary, our finding underlines the uncertain aetiology of erysipelas, and though it does not contradict erysipelas as primarily a streptococcal infection [1–5, 8–10], it demonstrates that other streptococci also can cause this condition in predisposed individuals.

## 4. Conclusions

Erysipelas is a common skin infection worldwide; however, its bacterial aetiology is still poorly understood. To our knowledge, these are the first episodes described of erysipelas caused by bacteria of the *S. mitis* group. Interestingly, our results indicate that two different species of this group, *S. mitis* and *S. oralis*, caused erysipelas, and it is remarkable that the clinical pattern has recurred on seven occasions during the last decade.

## Supplementary Materials

Supplementary Materials Table S1: MALDI-TOF MS identification and antibiotic susceptibility profile of isolated *S. mitis* and *S. oralis* isolates.
