# Supplementary material for: A Case of Recurrent Erysipelas Caused by Streptococcus mitis Group
Source: Case Rep Infect Dis. 2018 May 30;2018:5156085. doi: 10.1155/2018/5156085 (PMC6029475; doi:10.1155/2018/5156085)
Supplement: Supplementary Materials — Table S1: MALDI-TOF MS identification and antibiotic susceptibility profile of isolated S. mitis and S. oralis isolates. [file 5156085.f1.pdf]

Case Report in Infectious Diseases
Volume 2018, Article ID 5156085
https://doi.org/10.1155/2018/5156085
Case Report

A Case of Recurrent Erysipelas Caused by Streptococcus mitis Group

David Nygren, Bo Nilson, and Magnus Rasmussen

Supplement Table S1 MALDI-TOF MS identification and antibiotic susceptibility profile of isolated *S. mitis* and *S. oralis* isolates

	**MALDI Biotyper**[a]							**Specific peaks for *S. pneumoniae*, *S. mitis*, and *S. oralis*** [c]					**Antibiotic susceptibility profile**				
	**log (score)**		**list (score)**[b]					**m/z value**					**penicillin**	**vancomycin**	**gentamicin**	**clindamycin**	**optochin**
**Strain (Isolate no)**	**Best match**	**score value**	**p**	**m**	**o**	**sp**	**Best match**	**4964.3**[d]	**5297.6**[e]	**5822.5**[e]	**6839.1**[f]	**6888.9**[d]	**MIC (mg/L)**	**MIC (mg/L)**	**MIC (mg/L)**	**SIR**[g]	**SIR**[g]
15-1 (1)	*S. mitis*	2.28	2	92	26	0	*S. mitis*	-	-	-	+	-	0.064	0.25	2	S	R
17-1 (2)	*S. mitis*	2.51	0	90	43	0	*S. mitis*	-	-	-	+	-	0.125	0.5	2	S	R
17-2 (3)	*S. oralis*	2.48	0	11	113	0	*S. oralis*	-	-	+	+	-	0.125	1	16	S	R

[a] Bruker MALDI Biotyper database version 7311

[b] The list (score) is defined as the sum of weighted log (scores) for each single species according to Harju *et al* (20). p, *S. pneumoniae* ; m, *S. mitis* ; o, *S. oralis* ; sp., any other species in the MALDI Biotyper database.

[c] Specific peaks according to Marin *et al* (21)

[d] Peak associated with *S. pneumoniae*

[e] Peak associated with *S. oralis*

[f] Peak associated with *S. mitis* and *S. oralis*

[g] S, susceptibile; R, resistant
